# Interleukin-22 Alleviated Palmitate-Induced Endoplasmic Reticulum Stress in INS-1 Cells through Activation of Autophagy

**DOI:** 10.1371/journal.pone.0146818

**Published:** 2016-01-19

**Authors:** Minling Hu, Shuangli Yang, Li Yang, Yanzhen Cheng, Hua Zhang

**Affiliations:** Department of Endocrinology, Zhujiang Hospital of Southern Medical University, Guangzhou, P. R. China; Duke University Medical Center, UNITED STATES

## Abstract

**Objective:**

Stimulation with saturated fatty acids has been shown to induce oxidative stress and endoplasmic reticulum (ER) stress in β cells and has been recognized as an important component of the pathogenesis of type 2 diabetes (T2D). Interleukin-22 (IL-22) plays a critical role in preventing β cells from oxidative and ER stress, and autophagy is associated with the survival and function of β cells. However, whether IL-22 alleviates cellular stress through activation of autophagy is unclear. In this study, we investigated the effects of IL-22 on rat insulin-secreting cells and the mechanisms underlying IL-22 and lipotoxicity-induced oxidative and ER stress *in vitro*.

**Methods:**

The levels of reactive oxygen species (ROS) were detected by flow cytometry and fluorescence microscopy. The protein expression of glucose-regulated protein 78 (GRP78), C/EBP homologous protein (CHOP), microtubule-associated protein light chain 3B (LC3B) and Bcl-2-interacting myosin-like coiled-coil protein (Beclin-1) were evaluated by western blot. Transmission electron microscopy was utilized to observe the process of autophagy.

**Results:**

Palmitate induced increased levels of ROS and the overexpression of GRP78 and CHOP, whereas these effects were partly reversed by treatment with IL-22. Furthermore, IL-22 upregulated the protein expression of Beclin-1 and the conversion of LC3B-I to LC3B-II. Moreover, the aforementioned effects were partly suppressed by treating cells with 3-methyladenine (3-MA), an autophagy inhibitor.

**Conclusions:**

Our results suggest that IL-22 alleviated the oxidative and ER stress induced by palmitate, which was likely mediated by autophagy. These findings could facilitate the development of novel therapeutic strategies to suppress the progression of T2D.

## Introduction

Diabetes mellitus, one of the most common non-communicable diseases, threatens 8.3% of the global adult population suggesting a widespread public health epidemic. Approximately 382 million people had diabetes mellitus in 2013 and the net increase will reach 55% in 2035 [1]. Indeed, diabetes mellitus, which is accompanied by cardiovascular and atherosclerotic complications, has become a global economic burden. Moreover, type 2 diabetes (T2D) accounts for nearly 90% of all diabetes cases.

T2D is characterized by dysfunctional pancreatic β cells and insulin resistance and is caused by transitions in lifestyle and dietary habits, ageing of the population and urbanization in the setting of a genetically predisposed environment [2]. Because β cells continuously produce and secrete insulin, their endoplasmic reticulum (ER) has a high capacity for protein synthesis and folding, which in turn makes them particularly prone to develop stress when faced with the high protein-folding burden of proinsulin biosynthesis [3]. Considerable evidence indicates that ER stress, which occurs as a result of glucolipotoxicity, hypoxia and the accumulation of unfolded proteins in metabolic organs [4], contributes to progressive β cells dysfunction and loss in T2D [5, 6]. In fact, exposure to a high level of saturated fatty acids is known to promote ER stress by depleting ER calcium stores [7], but it is also a risk factor for the development of T2D. Recently, Hasnain et al. demonstrated that IL-22 is a powerful endogenous paracrine suppressor of oxidative and ER stress in pancreatic islets, regardless of whether the stress is induced by lipids, inflammatory cytokines or environmental reactive oxygen species (ROS) [8].

As a cytokine of the IL-10 cytokine family, IL-22 is predominantly expressed by innate lymphoid cells and activated T helper type 17 (TH17) and TH22 cells. IL-22 is expressed in a broad array of tissues, including the intestines, lung, liver, kidney, thymus, pancreas, and skin [9]. Numerous studies have shown that IL-22 mediates a physiologic response to repair local tissue damage in experimental models such as hepatitis, pancreatitis, colitis, and thymic injury [10, 11]. Controversially, IL-22 may contribute to pathophysiologic inflammation, along with IL-1, IL-6, IL-8, IL-11, G-CSF, and LPS binding protein [11]. In addition to the production at the site of inflammation, increasing evidence indicates that IL-22 improves insulin sensitivity and lipid metabolism in diabetes [12] and protects β cells from oxidative and ER stress, resulting in improved glycemic control [8]. The benefit effect of IL-22 in metabolism opens new avenues for novel therapy of metabolic diseases, however, the mechanism by which IL-22 alleviates oxidative and ER stress in pancreatic islets has not been definitively elucidated.

Autophagy, a conserved self-digestion process involved in cell growth, development, and homeostasis, plays an important role in maintaining the balance between the synthesis, degradation and subsequent recycling of cellular components [13]. Under various types of cellular stress, autophagy can act as an important mechanism [14] to promote cell survival and counteract the death of apoptotic cells [15]. Furthermore, autophagy is also responsible for removing damaged or redundant components of the ER, and exerts a protective effect against ER stress [16]. Accumulating studies suggest that autophagy plays a role in ameliorating ER stress in β cells during lipotoxicity, resulting in the maintenance of homeostatic control [17].

On the basis of these observations, in the present study, we investigated whether IL-22 alleviates oxidative and ER stress in INS-1 cells, and whether this process is mediated by autophagy.

## Materials and Methods

### Culture of INS-1 cells

INS-1 cells[18] (purchased from Life Technologies, Grand Island, NY) were cultured in RPMI-1640 supplemented with 10% heat-inactivated fetal bovine serum (FBS), 1 mM sodium pyruvate, 10 mM HEPES, 50 μM 2-mercaptoethanol, 100U/ml penicillin and 100 μg/ml streptomycin and were maintained at 37°C in a humidified atmosphere containing 5% CO_2_. The cells were trypsinized using 0.25% trypsin-EDTA and were then incubated in 6-well plates until they reached approximately 80% confluence. All experiments were performed using differentiated INS-1 cells that were 70%-80% confluent.

### Palmitic acid solutions

Palmitic acid (Sigma-Aldrich, Milano, Italy) was dissolved at 70°C in 0.1 M NaOH to obtain a 100 mM stock solution. A 5% (w/v) solution of FFA-free bovine serum albumin (BSA) (Sigma-Aldrich, Milano, Italy) was prepared in serum-free RPMI medium. The two aforementioned solutions were suitably combined to a PA/BSA mixture of 0.5 mM and the mixture was conveniently further diluted in RPMI to obtain the required final concentrations.

### ROS measurement

ROS production was determined by the redox-sensitive fluorescent dye DCFH-DA (Sigma-Aldrich, Milano, Italy). After treatment, cells were washed twice with PBS and then treated with 10 μmol/L DCFH-DA at 37°C for 20–30 min. Subsequently, the DCFH-DA-stained cells were washed three times in PBS, and then resuspended in PBS. To estimate the relative ROS accumulation, the fluorescence intensity was determined by flow cytometry (Becton Dickinson Verse, San Jose, CA, USA) and fluorescence microscopy (Zeiss EM-109, Jena, Germany).

### Western-blot analysis

The expression and phosphorylation level of target proteins were detected by western-blot analysis. Cells were lysed with RIPA lysis buffer containing a complete protease inhibitor mixture and a protein phosphatase inhibitor. After protein extraction, the cell lysates were separated by sodium dodecyl sulfate polyacrylamide gel electrophoresis and transferred to PVDF membranes. Nonspecific binding was blocked using 5% BSA, and the membranes were then incubated with specific primary antibodies overnight at 4°C. After incubating the membrane with the appropriate secondary antibodies conjugated to horseradish peroxidase, Super Enhanced chemiluminescence detection reagents were used to detect the specific bands. The immunoblots were quantified by densitometric analysis using Quantity One software (Bio-Rad, Hercules, USA). The following primary antibodies (1:1000) were used: anti-GRP78, anti-CHOP, anti-Beclin-1 (Santa Cruz Biotechnology, Santa Cruz, CA, USA), and anti-LC3B (Cell Signaling Technology, Danvers, MA, USA). GAPDH was used as an internal control.

### Immunofluorescence assay

After treatment, the cells were washed twice with PBS and fixed with 4% paraformaldehyde for 15–20 min. Then, the cells were permeabilized with 0.5% Triton X-100 in PBS for 10 min and blocked with 3.5% normal goat serum at room temperature for 1 h. After incubation with specific primary antibody overnight at 4°C, slides were stained with FITC-labeled secondary antibody for 1 h at room temperature and subsequently stained nuclei with DAPI. Images were captured with a laser confocal microscope (FV10-ASW, Olympus, Tokyo, Japan).

### Transmission electron microscopy (EM)

Cells were fixed with 2.5% glutaraldehyde for 2 h and washed with PBS three times. After immersion in 1% osmium tetroxide for 3 h and rinsing with PBS three times, the cells were immersed at 4°C in sequential 50, 70 and 90% ethanol for 15 min. The cells were then immersed in 90% ethanol and a 90% acetone solution for 15 min and were then immersed in 90% acetone for 15 min. Then, the cells were dehydrated with 100% acetone three times at room temperature. After being treated with 100% acetone plus embedding solution (2:1) for 3 h and then 100% acetone plus embedding solution (1:2) overnight, the cells were treated with 100% embedding solution for 3 h. Then, the specimens were cured and sliced to a thickness of 50 nm. Finally, the specimens were double stained with 3% uranyl acetate and lead citrate and were then observed using a transmission electron microscope (EM902A, Carl Zeiss MicroImaging GmbH, Germany).

### Statistical analysis

All experiments were conducted in triplicate. The results were expressed as the means ± SEM. The one-way ANOVA method was conducted to compare multiple groups. Two groups were compared using the LSD method; when the assumption of equal variance did not hold, Dunnett’s T3 method was used. Differences were considered statistically significant at *P* <0.05. Statistical analyses were conducted using SPSS 20.0 software.

## Results

### Palmitate induced oxidative stress in INS-1 cells

**Fig 1:**
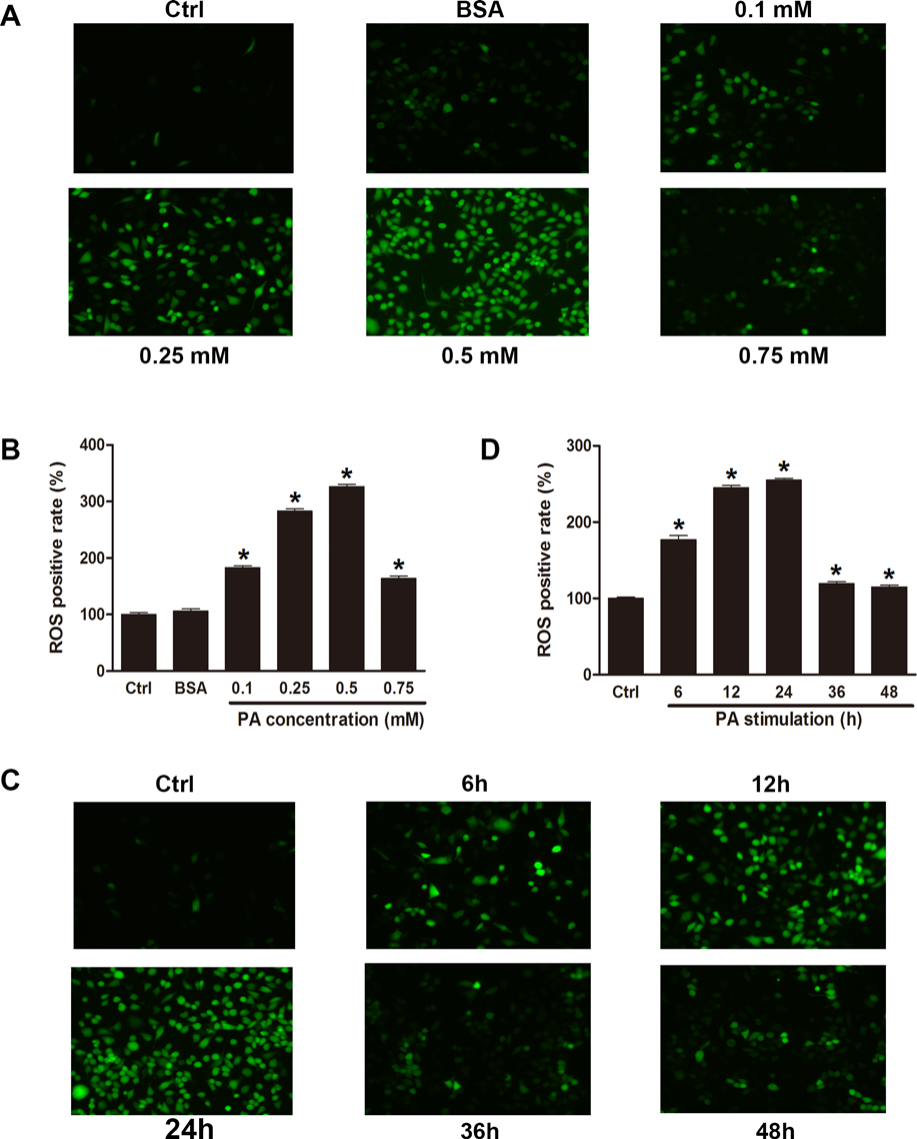
Palmitate induced oxidative stress in INS-1 cells. INS-1 cells were treated with control medium, FFA-free BSA (0.5 mol/ml), or indicated concentration of palmitate 24 h or 0.5 mM of palmitate for indicated times. The results showed that palmitate increased the level of ROS in INS-1 cells, which was measured by flow cytometry (B, D) and fluorescence microscopy (A, C). Data are expressed as means ± SEM of 3 independent experiments; *P < 0.05 vs. control.

Excessive ROS has been proposed to be implicated in impaired insulin resistance and the activation of the β-cell apoptotic pathway [19]. To investigate whether palmitate induced oxidative stress in INS-1 cells, we measured the ROS production by fluorescence microscopy and flow cytomety in INS-1 cells. Treatment with palmitate increased the ROS level in a dose- and time- dependent manner (Fig 1). However, the ROS level decreased in the 0.75 mM PA-treatment, 36-h PA-treatment and 48-h PA-treatment groups, perhaps because the cells were undergoing apoptosis during these conditions. Furthermore, this effect was not observed in either the control group or the BSA group. These results indicated that the increase in the ROS level was associated with palmitate. Collectively, these data suggested that palmitate induced oxidative stress in INS-1 cells.

### Palmitate induced ER stress in INS-1 cells through ROS induction

**Fig 2:**
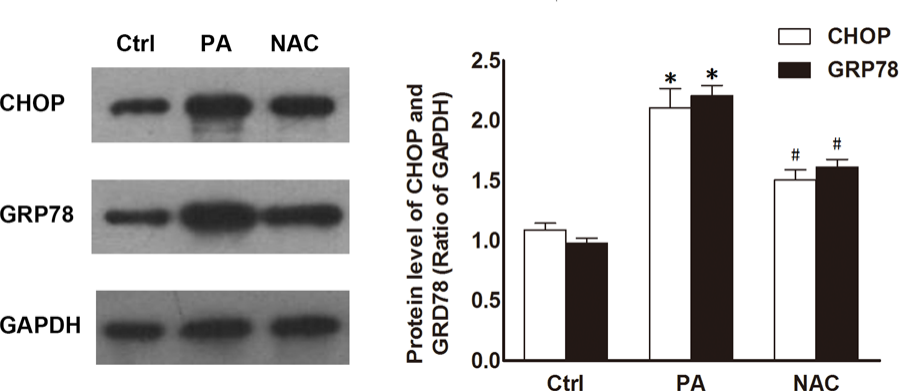
Palmitate induced ER stress in INS-1 cells via induction of ROS. INS-1 cells were treated with control medium or 0.5 mM of palmitate 24 h in the presence or absence of NAC (500 μmol/ml). The western-blotting showed that palmitate-induced overexpression of GRP78 and CHOP at protein levels were partly inhibited by NAC. Data are expressed as means ± SEM of 3 independent experiments; *P < 0.05 vs. control, #*P* < 0.05 vs. palmitate-treatment group.

Several studies have shown that the generation of ROS can trigger ER stress because protein folding is redox-dependent [20]; this process is involved in β-cell dysfunction and apoptosis [21]. Glucose-regulated protein 78 (GRP78) consequently increases the protein folding capacity of the ER and functions during ER stress, whereas C/EBP homologous protein (CHOP) is a key initiating factor in ER stress-related cell death. These represent 2 distinct mechanisms that are induced during ER stress and are thus regarded as markers of ER stress [22, 23]. To investigate the role of ROS in palmitate-induced ER stress in INS-1 cells, we examined the expression of GRP78 and CHOP in cells treated with or without palmitate and a ROS scavenger (n-acetyl-cysteine, NAC). Compared with the levels in the control cells, palmitate-induced overexpression of GRP78 and CHOP were partly reversed when cells were treated with palmitate and the ROS scavenger (Fig 2). Taken together, these results indicated that palmitate-induced ER stress in INS-1 cells was mediated by the accumulation of ROS.

### IL-22 alleviated the oxidative and ER stress induced by palmitate in INS-1 cells

**Fig 3:**
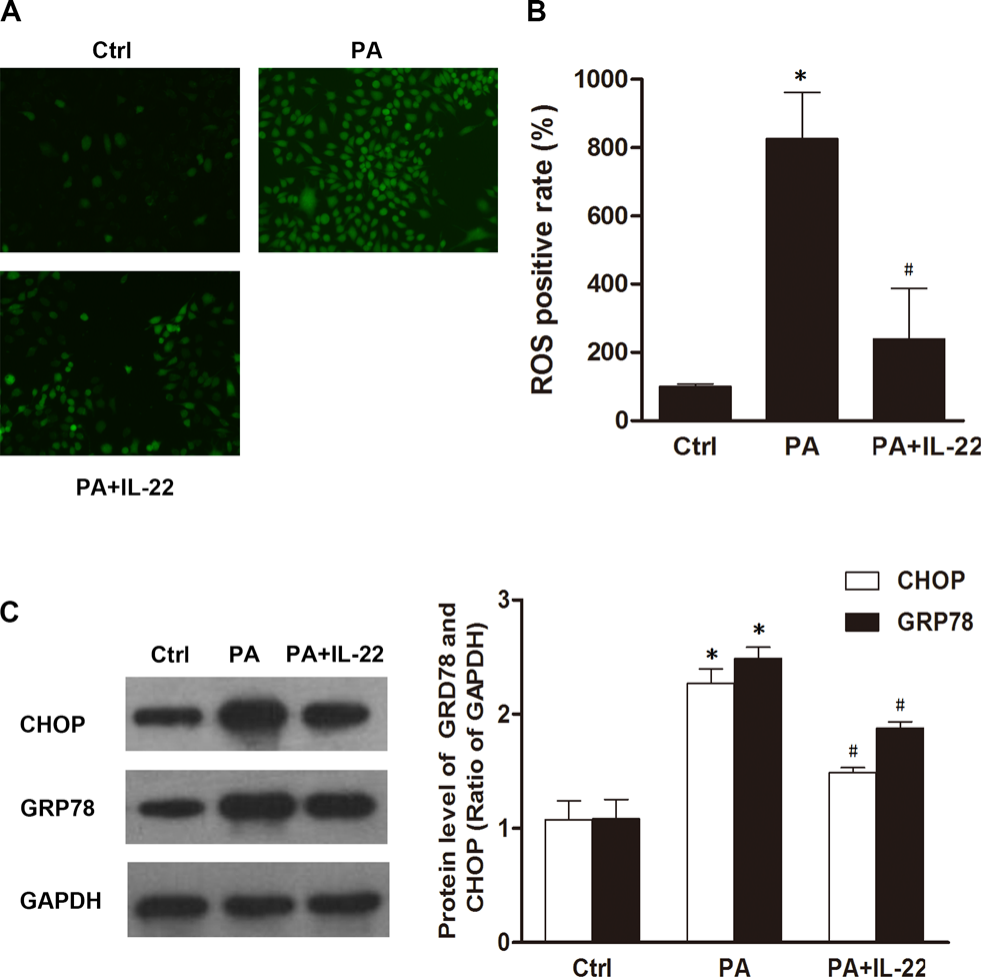
IL-22 alleviated palmitate-induced oxidative and ER stress in INS-1 cells. INS-1 cells were incubated with control medium, 0.5 mM of palmitate 24 h in the presence or absence of IL-22 (50 ng/ml). (A, B): Flow cytometry and fluorescence microscopy results showing that palmitate-induced increases in the level of ROS were partly reversed by IL-22. (C, D): Wester-blotting results showing that Il-22 downregulated the expression of GRP78 and CHOP induced by palmitate at the protein level. Data are expressed as means ± SEM of 3 independent experiments; *P < 0.05 vs. control, #*P* < 0.05 vs. palmitate-treatment group.

As a pro-inflammatory cytokine mediating crosstalk between the immune system and host cells, IL-22 has been proposed to regulate β cell insulin biosynthesis and secretion via the control of oxidative stress and ER stress [8]. Thus, to determine whether IL-22 alleviated palmitate-induced oxidative and ER stress, we measured the level of ROS and the expression of GRP78 and CHOP. The ROS level and the protein levels of GRP78 and CHOP were significantly increased in palmitate-treated INS-1 cells, and these effects were partly suppressed after treatment with IL-22 (Fig 3). Collectively, these data suggested that IL-22 alleviated the oxidative and ER stress induced by palmitate.

### IL-22 induced autophagy in palmitate-treated INS-1 cells

**Fig 4:**
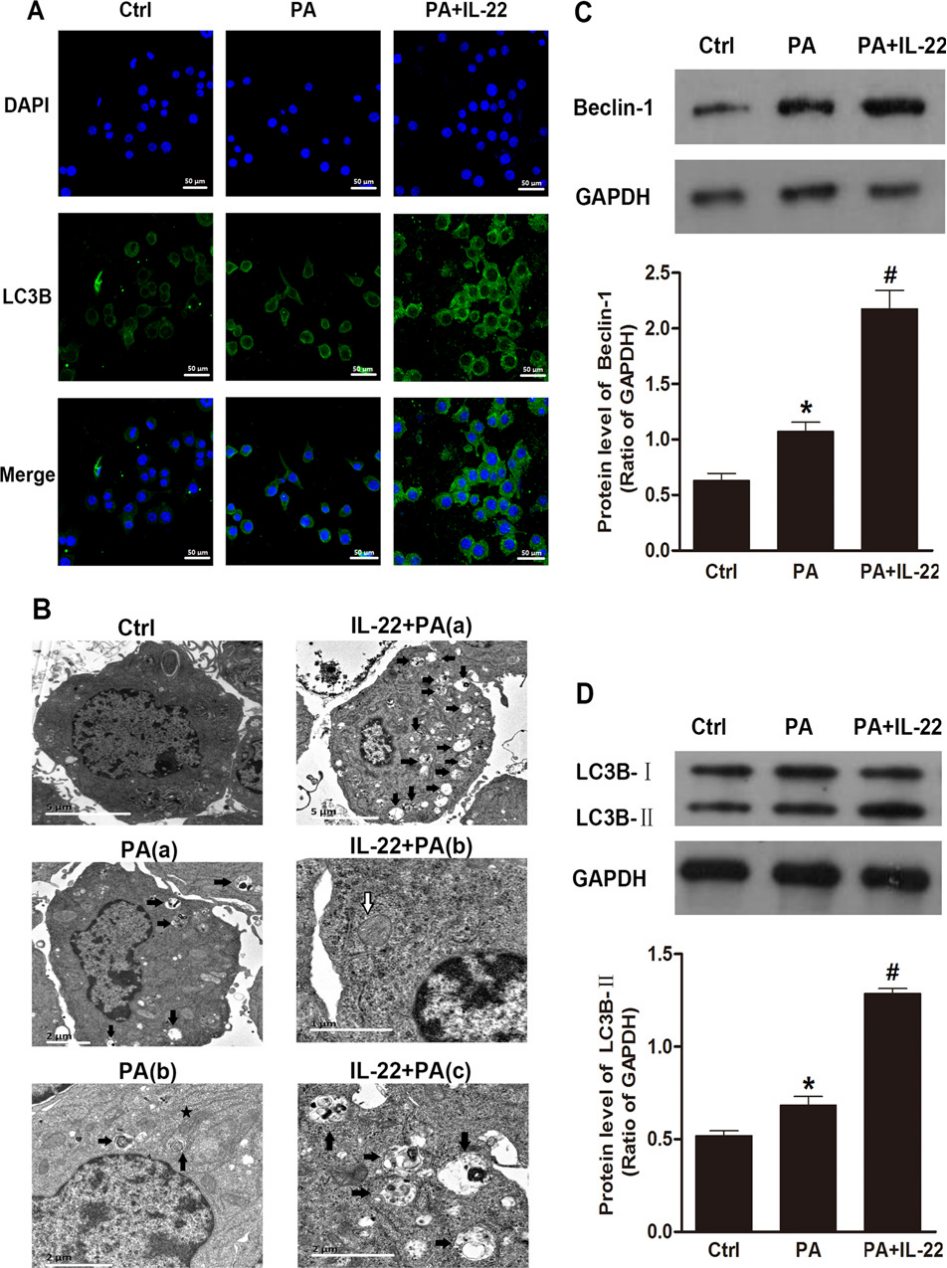
IL-22 induced autophagy in cultured INS-1 cells. INS-1 cells were cultured as control cells, were cultured with 0.5 mM palmitate 24 h in the presence or absence of IL-22 (50 ng/ml). The results of western-blotting (C, D) showed that IL-22 induced the overexpression of Beclin-1 and the conversion of LC3B-I to LC3B-II at the protein level following the treatment of palmitate. The results of immunofluorescence assay (A) and electron microscopy analysis (B) showed that IL-22 increased the puncta of LC3B (Scale bars, 50 μm) and the number of typical autophagosome and autophagolysosome during palmitate (autophagosome, white arrowheads; autophagolysosome, black arrowheads; ER cisterna, black star; Scale bars, Ctrl: 5 μm; PA(a) and (b): 2 μm; PA+IL-22(a): 5 μm; PA+IL-22(b): 1 μm; PA+IL-22(c): 2 μm). Data are expressed as means ± SEM of 3 independent experiments; *P < 0.05 vs. control, #*P* < 0.05 vs. palmitate-treatment group.

As an adaptive response, autophagy provides cells with a defensive mechanism to balance ER stress [16]. Activated by Atg7, microtubule-associated protein light chain 3 I (LC3-I) is modified to a membrane-bound form, LC3-II, which is essential for the dynamic process of autophagosome formation [24]. Bcl-2-interacting myosin-like coiled-coil protein (Beclin 1) regulates the autophagic process by promoting the generation of phosphatidylinositol 3-phosphate (PI3P) and interacts with various proteins that orchestrate autophagosome formation [25]. Thus, the two proteins are involved in autophagosome formation, which has been hypothesized to be the key regulator and indicator of autophagy [26]. Our results showed that treatment with palmitate and IL-22 upregulated the protein level of Beclin-1 and the conversion of LC3B-I to LC3B-II in INS-1 cells, both of which were significantly higher than that in the palmitate-treatment group (Fig 4C & 4D). Furthermore, electron microscopy analysis showed a marked increase in the number of typical autophagosomes, characterized by double-membraned vacuoles engulfing cytoplasmic structures, as well as autolysosomes, which are recognized as single-membrane vacuole structures containing high-density materials, in INS-1 cells treated with palmitate and IL-22 compared with palmitate-treated cells (Fig 4B). Moreover, increased LC3B puncta were found in INS-1 cells treated with palmitate and IL-22, compared with the palmitate-treatment group (Fig 4A). Taken together, these results indicated that IL-22 activated autophagy in palmitate-treated INS-1 cells.

### IL-22 alleviated oxidative and ER stress in palmitate-treated INS-1 cells through the activation of autophagy

**Fig 5:**
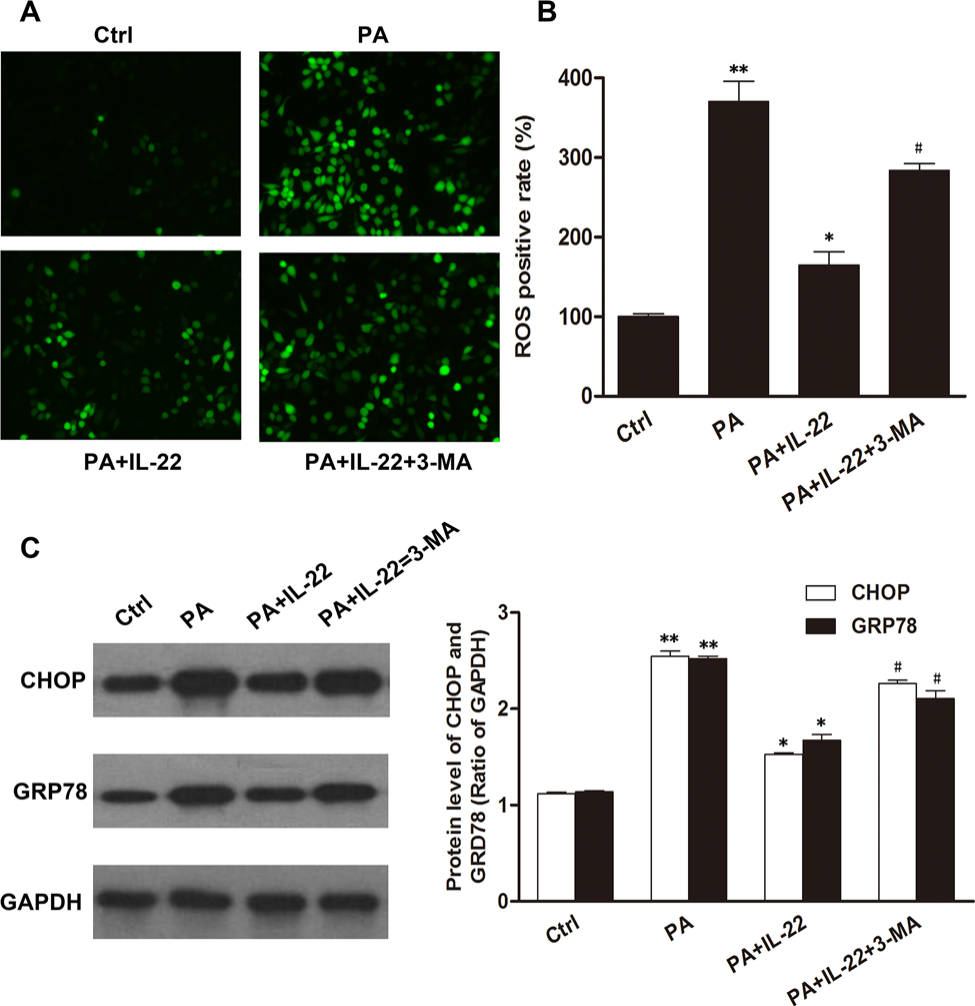
IL-22 protected INS-1 cells from palmitate-induced oxidative and ER stress, which was mediated by the activation of autophagy. INS-1 cells were treated with control medium, 0.5 mM of palmitate or 50 ng/ml of IL-22 24 h in the presence or absence of 3-MA. Palmitate-induced increases in the level of ROS (A, B) and the protein expression of GRP78 and CHOP (C, D) were partly reversed by IL-22, but these effects of palmitate were reproduced in cells treated with 3-MA. Data are expressed as means ± SEM of 3 independent experiments; **P < 0.05 vs. control, *P < 0.05 vs. palmitate-treatment group, #*P* < 0.05 vs. palmitate plus IL-22-treatment group.

To examine the role of autophagy in palmitate-induced oxidative and ER stress in INS-1 cells, we measured the accumulation of ROS and the expression of GRP78 and CHOP in the presence or absence of palmitate and an autophagy inhibitor (3-methyladenine, 3-MA). Our results showed that palmitate induced an increase in the ROS level and overexpression of GRP78 and CHOP compared with the control group; and these effects were partly suppressed after treatment with IL-22 (Fig 5). However, the downregulation of the ROS level and the protein levels of GRP78 and CHOP by treatment with IL-22 was partly reversed by 3-MA. Collectively, these data suggested that IL-22 alleviated palmitate-induced oxidative and ER stress in INS-1 cells through the activation of autophagy.

## Discussion

In the present study, we demonstrated that IL-22 protected rat insulinoma cells from palmitate-induced oxidative and ER stress and that these effects were mediated by autophagy.

As a contributor to metabolic interference and the cellular stress responses, chronic lipid accumulation plays an essential role in pancreatic β-cell dysfunction, which is characterized by impaired glucose-stimulated insulin secretion (GSIS) and increased apoptosis. It has been shown that lipotoxicity increased the production of ROS and oxidative stress, eventually leading to apoptosis and impaired insulin secretion, in the β cells of rat or human islets [27, 28]. As the hub for insulin synthesis and secretion, β cells are sensitive to ER stress. Moreover, elevated circulating saturated fatty acid levels could trigger ER stress in β cells through the depletion of ER calcium stores and a reduction in the ER capacity for other processes [29]. In accordance with previous studies, our results suggest that oxidative stress was involved in the pathway through which palmitate induced ER stress, which agree with evidence indicating that palmitate aggravates ER load via the accumulation of ROS [30]. Based on this evidence, we can conclude that palmitate induces ER stress in INS-1 cells and that this effect is associated with oxidative stress.

The role of oxidative stress and ER stress in the pathophysiology of T2D is attracting increasing attention; however effective therapies linked to this process are lacking. Recently, two studies indicated that IL-22 could restore mucosal immunity in diabetes by alleviating oxidative and ER stress in β-cells, which restored metabolic homeostasis [8, 12]. As an unusual interleukin that induces the production of antibacterial proteins and specific chemokines, IL-22 has been shown to ameliorate pancreatitis, hepatitis, inflammatory bowel disease, ulcerative colitis and diabetic wounds [30–32]. With respect to islet cells, IL-22 could upregulate the expression of the Reg gene and in turn induce the regeneration and differentiation of β cells [33]. Moreover, the alleviation of oxidative stress and ER stress by IL-22-treatment could suppress chemokine production in islets, because ER stress leads to inflammatory signaling [8]. In this study, our results suggest that palmitate-induced oxidative and ER stress in INS-1 cells were attenuated by IL-22 treatment, which support the previous finding that IL-22 suppressed oxidative and ER stress in mouse and human β cells leading to restoration of insulin secretion [8], and is consistent with evidence suggesting that IL-22 was able to prevent or reverse obesity-induced glucose intolerance and insulin resistance [12]. Collectively, these data indicated that IL-22 alleviated oxidative and ER stress induced by palmitate in INS-1 cells.

Our results suggested that IL-22 activated autophagy in INS-1 cells during palmitate treatment. Autophagy, a ubiquitous catabolic pathway functions to re-allocate mutrient from less critical processes to essential progress required for survival, has been involved in the progression and development of T2D. Evidence that the modulation of autophagy may be beneficial to preserve β-cell mass and function is beginning to accumulate [34, 35]. In addition to its role in the regulation of β-cell survival and function, autophagy can also maintain β-cell secretory granules at an optimal state and address the overwhelming accumulation of misfolded or aggregated proteins induced by cellular stress. It is noteworthy that autophagy functions to regulate intracellular lipid stores, which implicates its functional relevance in metabolic syndrome [36]. Moreover, Sharma et al. demonstrated that GLP-1 reduced the fat load in hepatocytes by increasing the rate of autophagosome and autophagolysosome formation, i.e., autophagic flux [37]. Consistent with a previous study, autophagy has been reported to play a protective role in PA-induced cell death and alleviate the lipid burden in INS-1 cells [38]. Thus, we confirmed that palmitate induced autophagy in rat insulin-secreting cells, which is related to the pathogenesis of T2D.

In β cells, the constant stimulus to secrete large amounts of insulin to maintain glycemia leads to an increased protein-folding burden in the ER, which contributes to ER stress. ER stress, together with the oxidative stress induced by excessive mitochondrial generation of ROS, leads to β-cell dysfunction. Accumulating data suggests that autophagy protects stressed β cells by eliminating damaged organelles and misfolded proteins, notably proinsulin [14]. However, although studies have suggested cross-talk between ER stress and autophagy, the potential effect of autophagy on palmitate-induced oxidative and ER stress in INS-1 cells has not been previously investigated. Our results showed that IL-22 prevents β cells from palmitate-induced oxidative and ER stress through the activation of autophagy. In accordance with our finding, Jung et al. indicated that the impairment of autophagy caused insulin deficiency and hyperglycemia because of abnormal turnover and impaired function of cellular organelles [35]. In addition, Quan et al. has demonstrated that autophagy-deficient β-cells were more susceptible to ER stress induced by thapsigargin or lipids [39]. Collectively, these data indicated that IL-22 alleviated the oxidative and ER stress in INS-1 cells induced by palmitate, which was associated with the activation of autophagy.

Our results clearly indicated that IL-22 protected INS-1 cells from palmitate-induced oxidative and ER stress through the activation of autophagy, although the molecular mechanism of IL-22-induced activation of autophagy remains to be fully elucidated. Furthermore, the pathways linking ER stress and autophagy in β cells have not been fully elucidated. It has been reported that IL-22 induced the proliferation of keratinocytes mediated by the PI3K/Akt/mTOR signaling pathway [40], which is also involved in autophagy signaling. On the other hand, research in both cell lines and mouse models showed that ER stress diminished the PI3K/AKT/mTOR pathway via the hyperactivation of JNK [41], which contributed to autophagy activation in the early phase of ER stress. Further investigations must be conducted to determine whether these pathways are involved in the IL-22-induced autophagy mediated alleviation of oxidative and ER stress during palmitate stress.

In conclusion, our results demonstrate that IL-22 protected INS-1 cells from palmitate-induced oxidative and ER stress through the induction of autophagy. Importantly, IL-22 therapies that regulate the β cell stress response to lipotoxicity may have clinical applications as they could prevent or attenuate the progression of T2D.
